# Perceptions About Biosimilar Medicines Among Belgian Patients in the Ambulatory Care

**DOI:** 10.3389/fphar.2021.789640

**Published:** 2022-01-05

**Authors:** Yannick Vandenplas, Liese Barbier, Steven Simoens, Philippe Van Wilder, Arnold G. Vulto, Isabelle Huys

**Affiliations:** ^1^ Department of Pharmaceutical and Pharmacological Sciences, Clinical Pharmacology and Pharmacotherapy, KU Leuven, Leuven, Belgium; ^2^ Ecole de Santé Publique, Université Libre de Bruxelles (ULB), Brussels, Belgium; ^3^ Hospital Pharmacy, Erasmus University Medical Center, Rotterdam, Netherlands

**Keywords:** biological, biosimilar, Belgium, perception, knowledge, education, patient

## Abstract

**Background and objectives:** Biosimilar medicines have been on the European market for 15 years. Despite the extensive and positive experience with biosimilars across Europe, their uptake remains limited in Belgium. One of the possible factors limiting uptake in clinical practice is the inadequate understanding and lack of trust in biosimilars among patients. This study aimed to assess the level of knowledge and perceptions about biosimilar medicines among Belgian patients in the ambulatory care.

**Methods:** This study consisted of online questionnaires among Belgian patients in the ambulatory care (i.e., rheumatoid arthritis, ankylosing spondylitis, psoriatic arthritis, plaque psoriasis, Crohn’s disease, ulcerative colitis, diabetes mellitus type I and II). The results were collected between December 2020 and February 2021. The data were analyzed with descriptive and inferential statistics.

**Results:** In total, 657 patients across all disease areas of interest participated in this study. Only 38% of patients had heard of biosimilars before. Of those patients, most (58%) were aware that biosimilars are as safe and effective as their reference product. The vast majority of respondents (68%) would agree with transitioning to a biosimilar if their physician prescribed it, only 3% would never agree with a transition to a biosimilar. If a physician would propose to change their current originator biological therapy with its biosimilar, nearly all patients (95%) want their physician to explain the decision and inform them. For additional information about biosimilars, Belgian patients prefer brochures or folders (41%), or available resources on the internet (35%). Physicians were indicated as the preferred source of information (95%), followed by pharmacists (51%), academia (39%), and patient associations (35%). Most patients require information regarding the safety and efficacy (78%), price and reimbursement (64%), and the clinical development process (56%) of the biosimilar.

**Conclusion:** Belgian patients require information about biosimilar medicines. However, most patients are open and positive towards transitioning their current biological therapy with its biosimilar if sufficiently supported by their healthcare providers.

## 1 Introduction

Since 2021, biosimilar medicines have been on the European market for 15 years. Despite the extensive and positive experience with biosimilars, and the fact that from a scientific point of view the equivalence with their reference product should no longer be questioned, their uptake varies widely within Europe (Barbier et al., 2020a; IQVIA, 2020). High biosimilar market shares are observed in the United Kingdom, Denmark, Norway, and the Netherlands. Typically slower adopters such as Belgium, Poland, or Romania experience difficulties capturing the full potential of a more competitive market, generated by the market entry of biosimilars (Moorkens et al., 2020c; Vandenplas et al., 2021b). Expenditures on biologicals have been increasing year after year in Europe, with a growing proportion that has lost their market exclusivities and may therefore come under competition from biosimilars (IQVIA, 2020). The potential of biosimilars lies mainly in their contribution to more sustainable healthcare systems, by keeping biological medicines affordable and providing patients with more access to these therapies (Vulto, 2019).

Each country within the European Union (EU) has its own healthcare system. Therefore, countries with low biosimilar uptake have their own specific reasons for this (Ingrasciotta et al., 2015; Remuzat et al., 2017; Moorkens et al., 2019, 2020a; Lobo and Río-Álvarez, 2021; Van Wilder, 2021). However, general elements seem to be the lack of prescribers’ incentives, limited guidance and position of regulatory authorities about transitioning to biosimilars, the innovator’s reach, and the limited understanding of biosimilars among healthcare providers (HCP) and patients (Moorkens et al., 2016; Remuzat et al., 2017). The latter is an important and widely studied element. Several large-scale studies have already indicated limited knowledge and confidence about biosimilars among HCPs and patients across Europe (Leonard et al., 2019; Vandenplas et al., 2021a). Poor knowledge or trust in biosimilars among patients may hamper the acceptance, hence impede uptake of biosimilars (Pouillon et al., 2018; Barbier et al., 2020b). Moreover, patients who have more trust in their medicine are less prone to develop *nocebo* effects, possibly leading to treatment failure. This is of particular importance when transitioning from an originator biological to its biosimilar. Transitioning (or *switching*) in this particular context refers to exchanging the patient’s originator biological medicine with its biosimilar, upon the initiative of the prescribing physician (European Medicines Agency, 2017). The *nocebo* effect is described as the increase in side effects or symptoms associated with a negative attitude towards a given medicine (Kravvariti et al., 2018; Kristensen et al., 2018). Clinical studies have already shown that when patients are well informed about biosimilars through an enhanced communication strategy when transitioning, the acceptance and persistence is higher (Tweehuysen et al., 2018b; Tweehuysen et al., 2018a).

An increasing share of off-patent biologicals or biosimilars is dispensed in Belgian public pharmacies, or the ambulatory care setting. Biosimilars available in the Belgian ambulatory care include TNF-alpha inhibitors for the treatment of immune mediated inflammatory diseases (i.e., adalimumab, etanercept) and insulins for the treatment of diabetes mellitus (i.e., insulin glargine). In the coming years, several new biosimilars are expected to enter the Belgian ambulatory care, such as golimumab, certolizumab pegol, insulin aspart, and ustekinumab (GaBi online, 2019; Zorginstituut Nederland, 2020). An overview of all biosimilars available in the Belgian ambulatory care setting is provided in Supplementary Material. Other biosimilar products not mentioned in this list are exclusively dispensed in the Belgian hospital setting, such as trastuzumab, rituximab, infliximab, pegfilgrastim, and epoetin.

In Belgium, it has been suggested that the lack of trust among patients is one of the contributing factors to the low biosimilar usage (Lepage-Nefkens et al., 2013; Dylst et al., 2014; Moorkens et al., 2020c; Vandenplas et al., 2020). However, only one study has been conducted that specifically assessed the knowledge and perceptions of Belgian patients about biosimilars (van Overbeeke et al., 2017). This study found that the level of knowledge and trust in biosimilars is rather limited, but even more among physicians than patients. This particular questionnaire study was conducted in 2016 among patients with rheumatoid arthritis. However, the biosimilar landscape has evolved since then with new biosimilars that have entered the market in new therapeutic areas. Moreover, educational efforts have been done to increase the knowledge of patients during past years, such as the information campaign of the Belgian regulatory authority in 2018 (Federaal Agentschap voor Geneemiddelen en Gezondheidsproducten, 2018). As a result, the situation might have changed and perceptions among patients could have changed.

Therefore, the aim of this study was to elicit the views and perceptions of Belgian patients in the ambulatory care towards biosimilar medicines, and to clarify the information needs of patients regarding biosimilars. The focus on ambulatory care is particularly relevant in the context of the availability of intravenous and subcutaneous administration forms of biologicals and possible different injection devices. Because biologicals used in this setting are primarily administered subcutaneously, patients are more aware of a possible transition to a biosimilar. For this reason, perceptions and information needs about biosimilars in the ambulatory setting are of particular interest. Therapeutic areas where biosimilars are available in ambulatory care, thus dispensed in community pharmacies, are rheumatology, dermatology (i.e. psoriasis), gastroenterology (i.e. inflammatory bowel diseases), gynecology, and endocrinology (i.e. diabetes type I and II). Recently, biosimilars in the ambulatory setting are also available for enoxaparin sodium, which is used as coagulation prevention (e.g., after surgery).

## 2 Methods

This study consisted of online questionnaires that were distributed to Belgian patients between December 2020 and February 2021.

### 2.1 Recruitment

As stated above, eligible participants included Belgian patients diagnosed with diseases for which biosimilars are available in the ambulatory care setting (i.e., rheumatoid arthritis, ankylosing spondylitis, psoriatic arthritis, plaque psoriasis, Crohn’s disease, ulcerative colitis, diabetes mellitus type I and II). Patients were recruited through Belgian patient associations representing the different diseases of interest. Patient associations were chosen as the route via which the surveys were distributed for feasibility reasons. In this way, a large patient group could be reached and the anonymity of the participant remained preserved. All major patient associations, both representing French- and Dutch-speaking patients, agreed to contribute to this research (i.e. RA Liga, Reumanet, Psoriasis Liga Vlaanderen, Psoriasis Contact, CLAIR, Association Crohn-RCUH, CCV vzw, Diabetes Liga, Association du Diabète).

An invitation was sent to all participating patient organizations via e-mail, who distributed the invitation to their members (via their web page, e-mail, social media). This invitation included general information about the study and the link to participate to the study. The message included that only Belgian patients were eligible to participate and that no prior knowledge about biosimilar medicines was deemed necessary, in order to avoid selection bias. No informed consent was required in order to process personal data, since all data were collected anonymously. The study was approved by the Ethics Committee UZ/KU Leuven (S64703).

### 2.2 Survey Design and Conduct

Before the questionnaires were sent to participants, the surveys were piloted with a representative from each patient organization to ensure all questions were clear and the translations were correct. The pilot study only led to minor alterations to the survey to increase the understandability of the questions.

Questionnaires were developed in advance by the research team (YV and LB), with the following sections: characteristics, knowledge, and perceptions. In the characteristics section, questions were asked about the participants’ age, region, gender, education level, treatment type, and diagnosis. This part was followed by the second part with questions on patients’ knowledge about biosimilars. The third and final part on perceptions was the largest part and assessed the opinion of patients about switching, substitution, needs regarding transitioning, and overall informational needs. Most questions were close ended multiple-choice questions, where one or more answers could be selected. However, an answer option with an open text field was provided most of the time to capture all relevant insights. Surveys were translated into French and Dutch so all patients were able to participate in their language of preference. SurveyMonkey was used for the design of the online survey. The software allowed to ensure that the same respondent could not complete the survey more than once. The complete questionnaire can be found in Supplementary Material.

### 2.3 Data Analysis

All data were analyzed descriptively via Microsoft Excel software, after extraction from SurveyMonkey software. Differences between specific subgroups of interest (i.e., high and low educated patients, Belgian regions) were analyzed with inferential statistics using Statistica software (Version 14). For these subgroup analyses, the Fisher Exact test was performed to assess differences in proportions. The level of significance was set at 5%, meaning *p*-values lower than 0,05 were considered statistically significant. Because of some drop-out during the completion of the questionnaire, the answers to questions more towards the end of the survey were completed by a lower number of respondents. In each case, the analysis was based on all completed responses, including those of participants who did not complete the survey. The percentages in the responses were therefore based on the number of responses per question.

## 3 Results

### 3.1 Participants’ Characteristics

In total, 657 patients participated in this study. Most participants were 40 years or older (72%, *n* = 474). Patients were mainly female (66%, *n* = 436) and living in Flanders (63%, *n* = 416). Patients from all disease areas of interest participated in this study, with the majority of patients in the group inflammatory bowel diseases (31%, *n* = 205), rheumatological inflammatory diseases (23%, *n* = 149) and psoriatic disorders (23%, *n* = 149). Other participants were diagnosed with diabetes (18%, *n* = 117) or other chronic conditions (6%, *n* = 37).

Almost all participants have an education of secondary school or higher (98%, *n* = 641), with the majority having a higher education (65%, *n* = 430). In our sample, 62% (*n* = 407) of all patients indicated that they were currently treated with a biological medicine. Only a minority was currently using oral (37%, *n* = 243) or topical (21%, *n* = 138) treatments. All patient characteristics are summarized in Table 1.

**TABLE 1:** Demographic data of participating patient (N: Number of participants).

Patients’ demographics	N (%)
Age	
18–29 years	73 (11%)
30–39 years	110 (17%)
40–49 years	135 (21%)
50–59 years	146 (22%)
60 years or more	193 (29%)
Gender	
Male	220 (33%)
Female	436 (66%)
I prefer not to answer	1 (<1%)
Other	0 (0%)
Region	
Flanders	416 (63%)
Wallonia	64 (10%)
Brussels	177 (27%)
Diagnosis	
Plaque psoriasis	149 (23%)
Psoriatic arthritis	70 (11%)
Diabetes mellitus (Type I or II)	117 (18%)
Rheumatoid arthritis	62 (10%)
Ankylosing spondylitis	17 (3%)
Crohn’s disease	124 (19%)
Ulcerative colitis	81 (12%)
Other	37 (6%)
Education level	
No diploma or primary school	16 (2%)
Secondary school	211 (32%)
Non-university higher education	292 (44%)
University higher education	138 (21%)
Treatment type	
Local or topical treatment	138 (21%)
Oral treatment	243 (37%)
Biological treatment	407 (62%)
Total	657 (100%)

### 3.2 Knowledge About Biosimilar Medicines

Most patients indicated that they had not heard of biosimilars before (62%, *n* = 401). However, this proportion seems to be lower for Dutch-speaking patients (54%, *n* = 216), compared to French-speaking patients (73%, *n* = 185). To patients who indicated to have heard about biosimilars before, a follow-up question was asked regarding the source by which they were informed. Their physician (61%, *n* = 138), patient organization (41%, *n* = 93), and the internet (41%, *n* = 93) were the most common sources.

To patients who have heard of biosimilars, two separate questions were posed with several statements about biosimilars regarding their equivalence or differences with original biological medicines. For the first question, 52% (*n* = 116) of participants indicated that a biosimilar medicine is equivalent to its reference product in terms of clinical outcomes. Yet, 26% of participants (*n* = 59) had heard about biosimilars without knowing what it means exactly. In a second question, participants were asked whether biosimilars are more, less, or as effective and safe compared to their reference biological product. Most patients that had heard of biosimilars believe that biosimilars are as safe and effective as their reference product (58%, *n* = 131). Only 5% (*n* = 11) indicated that biosimilars are less effective, and 28% (*n* = 64) did not know. No statistically significant differences between higher and lower educated patients, nor between French- and Dutch-speaking patients, were found for this question (Cfr. Supplementary Material).

### 3.3 Perceptions and Opinions About Biosimilar Medicines

#### 3.3.1 Opinion on Transitioning and Substitution

Patients were asked whether they think their physician should be able to change their current originator biological therapy with its biosimilar. Overall, the majority of patients (53%, *n* = 284) indicated that their physician should be able to switch their therapy to a biosimilar. Only 14% (*n* = 75) of participants indicated that their physician should not be able to change their biological therapy with its biosimilar. For chemical medicines, 63% (*n* = 337) of the surveyed patients agreed with the physician being able to change an original small molecule with its generic. In addition, 14% (*n* = 72) did not agree with this.

Patients were asked the same question, but now whether their pharmacist should be allowed to change their current biological therapy with a biosimilar (i.e., pharmacy-level substitution). Most patients (55%, *n* = 298) disagreed that pharmacists should be able to transition their originator therapy with a biosimilar. Only 22% (*n* = 118) agreed with pharmacy level substitution for biological medicines, and 17% (*n* = 92) thinks this depends on the product class. For chemical medicines, 38% (*n* = 200) indicated that their pharmacist should be able to change their original chemical medicine with its generic. The largest group of patients (45%, *n* = 240) did not agree with pharmacists being able to substitute chemical medicines.

In subsequent questions, patients were asked whether or not they are currently being treated with a biological medicine. More specifically, they were asked whether they are treated with an originator biological or a biosimilar medicine. To further tailor the answers, follow-up questions were asked only to patients who indicated to be treated with an originator biological, or were not sure whether they are treated with an originator or biosimilar biological therapy.

**FIGURE 1:**
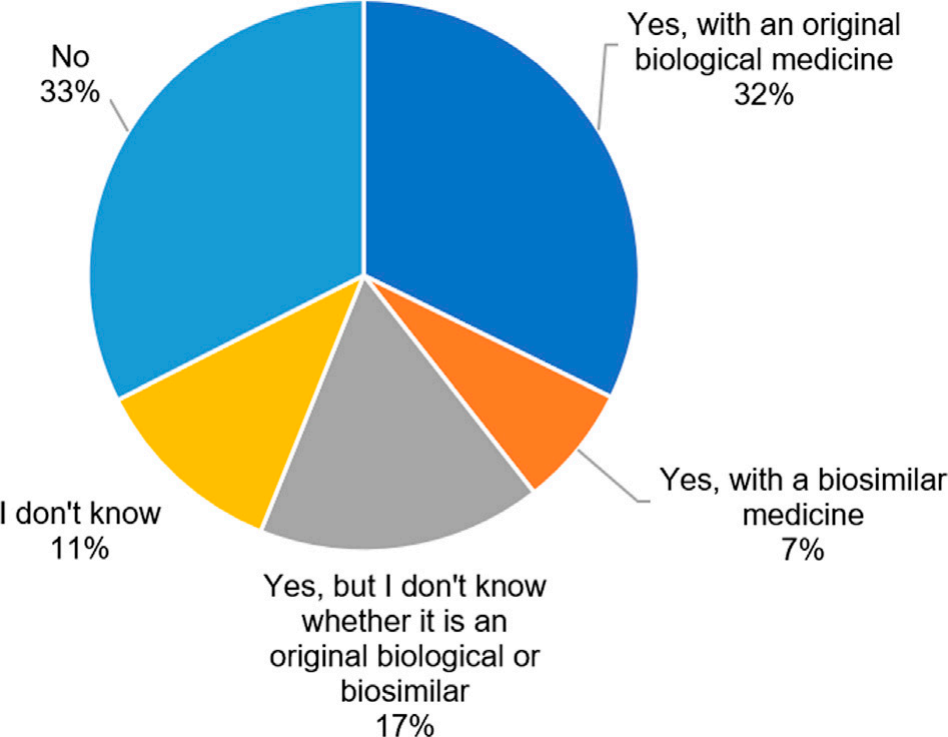
Are you currently being treated with a biological medicine? (N = 533, N: Number of participants).

The small majority of the respondents indicated to be treated with a biological (56%, *n* = 299), of which 58% (*n* = 172) were treated with originator biologicals and 13% (*n* = 38) with biosimilars. The remainder (30%, *n* = 89) indicated that they were using a biological, but did not know whether it was an originator or a biosimilar medicine (Figure 1).

**FIGURE 2:**
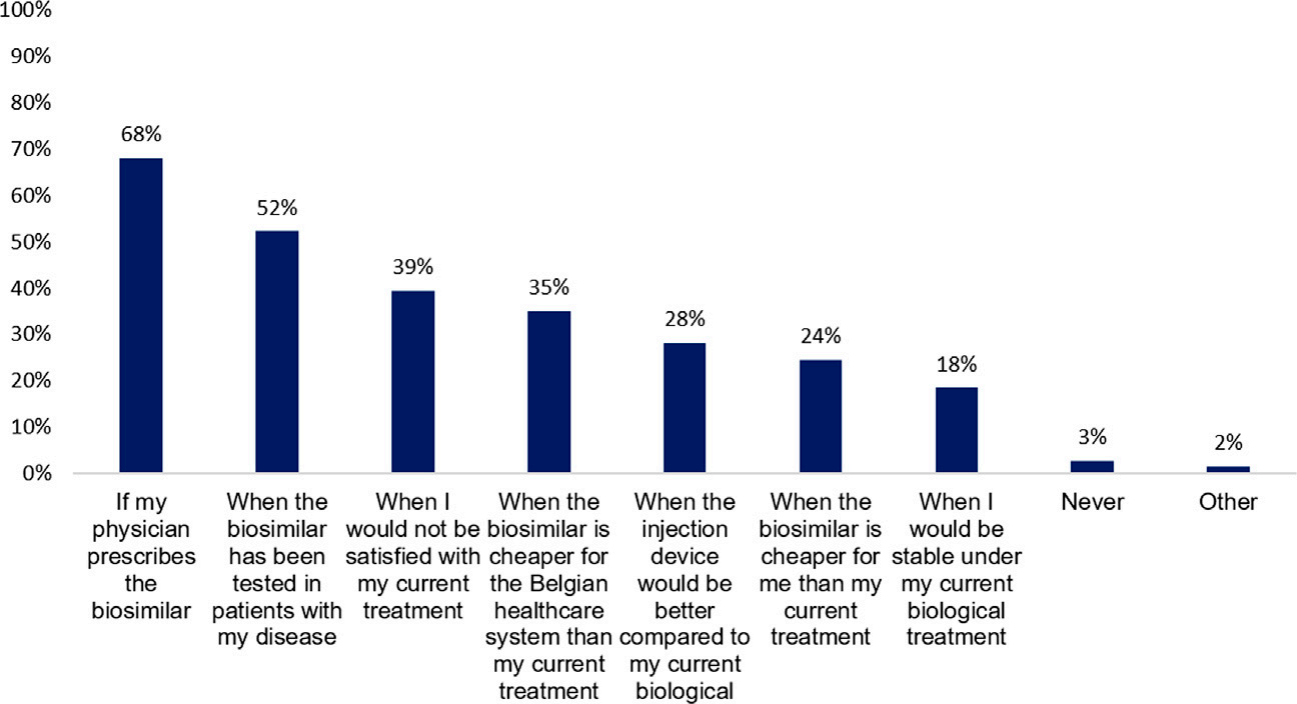
Under what circumstances would you change your current original biological therapy with its biosimilar? (N = 249, N: Number of participants).

To patients who indicated to be treated with an originator biological or who were unsure, questions were asked about under what circumstances they are willing to transition to a biosimilar. The vast majority of respondents (68%, *n* = 169) would agree with transitioning to a biosimilar if their physician prescribed it. Only 3% (*n* = 7) would never agree with a transition to a biosimilar. However, a large proportion (52%, *n* = 130) would only be willing to transition to a biosimilar that has been tested in their specific disease or when they are unsatisfied with their current treatment (39%, *n* = 98). Some patients would let it depend on a better injection device (28%, *n* = 70) or when the biosimilar is cheaper for the healthcare system (35%, *n* = 87) or themselves (24%, *n* = 61) (Figure 2). For this question, differences were assessed between higher and lower educated patients, as well as between French- and Dutch-speaking patients. No significant differences were observed for any of the statements between high and low educated participants. However, more Dutch-speaking patients would like the biosimilar to be tested in their specific disease (60 vs 38%, *p* = 0,00142) and would only transition to a biosimilar when they are not satisfied with their current originator biological treatment (46 vs 28%, *p* = 0,00698), compared to French-speaking patients (Cfr. Supplementary Material).

#### 3.3.2 Patients’ Needs Regarding Switching to a Biosimilar

Questions about patients’ needs regarding transitioning to a biosimilar were asked to patients receiving treatment with an originator biological medicine only. Participants were asked to indicate which questions they would ask their physician when she/he would propose to transition to a biosimilar. The majority would ask questions about the safety and efficacy of biosimilars (83%, *n* = 207) or ask their physician about the reasons why the switch is being made (72%, *n* = 178). Most patients would ask what the experiences of their physician are with changing from an originator therapy to its biosimilar (61%, *n* = 152).

**FIGURE 3:**
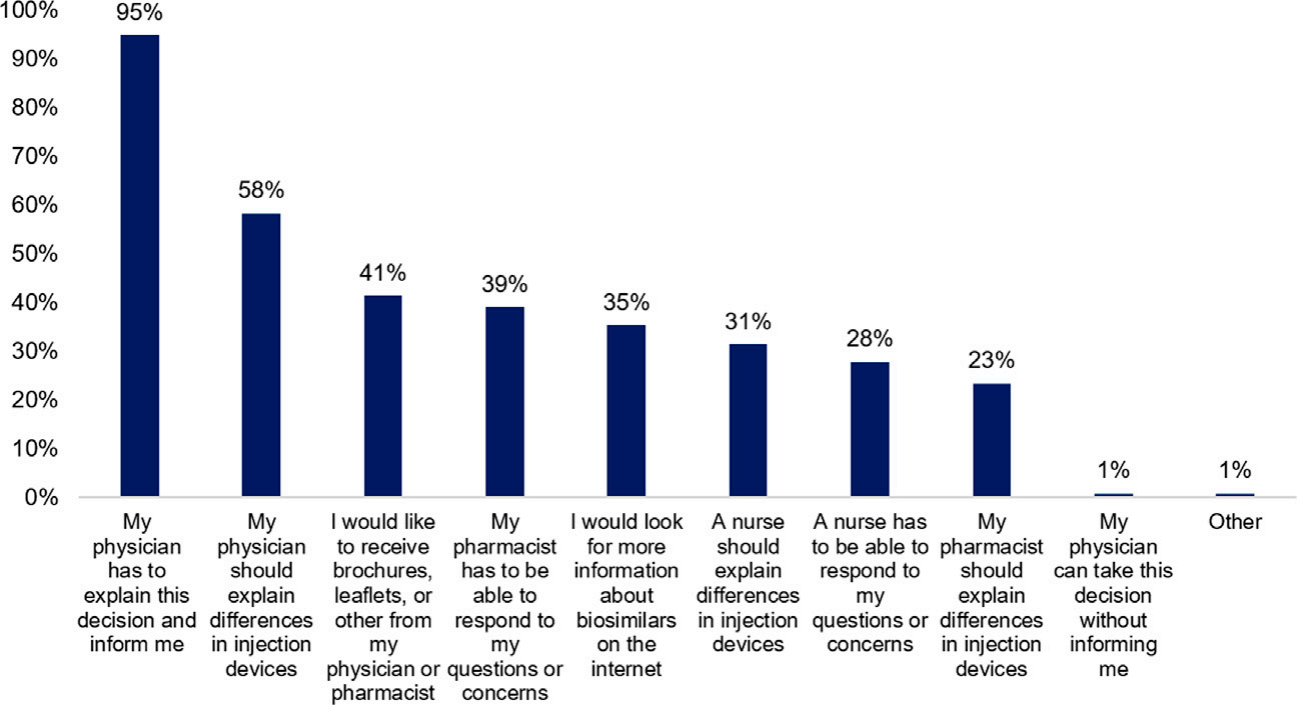
What kind of support do you expect when the decision has been made to change your current biological therapy with its biosimilar? (N = 249, N: Number of participants).

Subsequently, patients were asked to indicate what kind of support they require when switching. Almost all patients want their physician to explain the decision and inform them (95%, *n* = 236). Patients expect that their physician (58%, *n* = 145) or nurse (31%, *n* = 78) explains possible differences in injection devices. They prefer to receive additional information about biosimilars via brochures or folders (41%, *n* = 103) as well, or would look for information on the internet by themselves (35%, *n* = 88). Moreover, pharmacists (39%, *n* = 97) and nurses (28%, *n* = 69) should be able to respond to their questions or concerns after transitioning to a biosimilar (Figure 3).

#### 3.3.3 Patients’ Information Needs About Biosimilars

In the final subsection, patients were questioned on their information needs about biosimilar medicines in general. When asked what they would do when requiring more information about biosimilars, the majority of respondents would ask their physician (87%, *n* = 441). In addition, a large part of respondents would look for further information about biosimilars on the internet (64%, *n* = 322).

**FIGURE 4:**
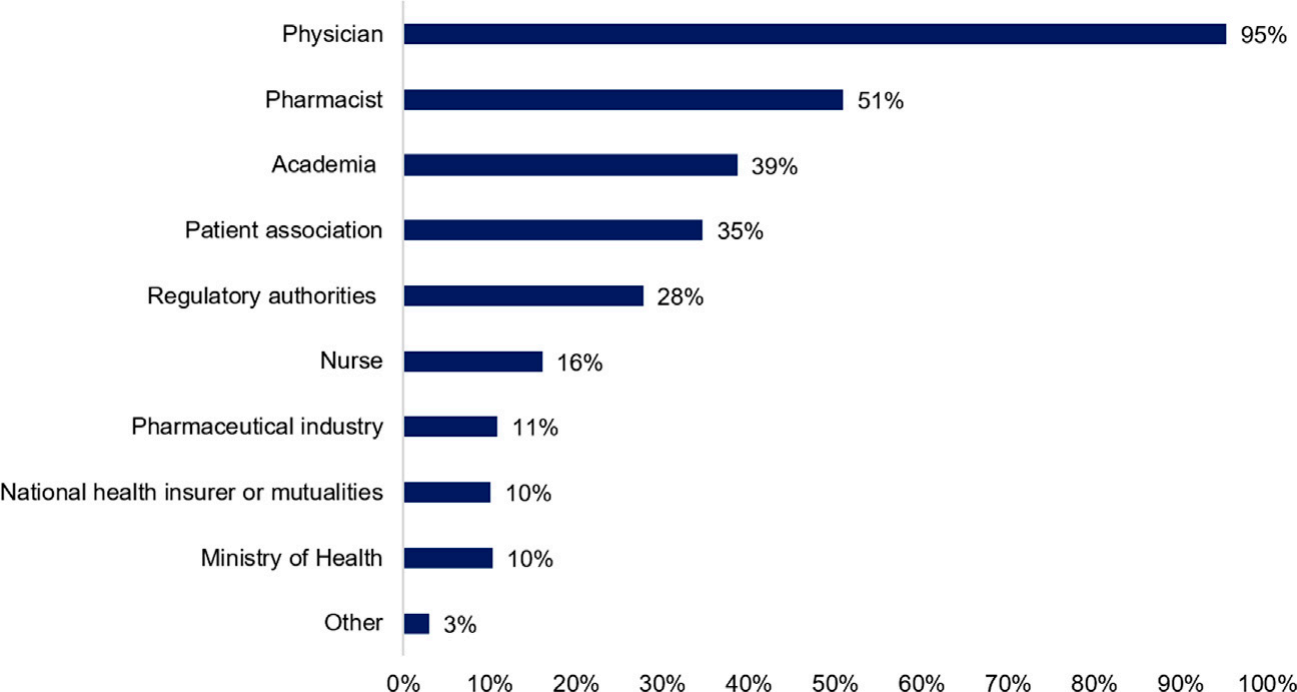
Whom do you consider a trusted source of information about biosimilar medicines? (N = 505, N: Number of participants).

Information about biosimilars can emerge from multiple sources. Therefore, patients were asked to indicate which sources they trust most. In accordance with earlier questions, physicians were indicated as the most trustworthy source of information (95%, *n* = 481), followed by pharmacists (51%, *n* = 257), academia (39%, *n* = 195), and patient associations (35%, *n* = 175). Noteworthy, only a minor part of participating patients indicated the governmental institutions such as the regulatory agency (28%, *n* = 140), ministry of health (10%, *n* = 52), and national health insurer (10%, *n* = 51) as a trusted source of information (Figure 4).

Most patients require information regarding biosimilar medicines about their safety and efficacy (78%, *n* = 369), their price and reimbursement (64%, *n* = 322), and the clinical development process of the biosimilar (56%, *n* = 284). In addition, patients showed interest in information about injection devices, the use of biosimilars (49%, *n* = 248), and their quality requirements (44%, *n* = 222).

## 4 Discussion

### 4.1 Knowledge About Biosimilars

In past years, several studies have already shown an inadequate understanding about biosimilar medicines among European patient groups across different indications (Jacobs et al., 2016; Aladul et al., 2017; Peyrin-Biroulet et al., 2017; van Overbeeke et al., 2017; Waller et al., 2017; Azevedo et al., 2018; Frantzen et al., 2019). However, most of these studies assessed this among the broader patient population. After all, it is only relevant to examine this in patients who could come into contact with biosimilars (Barbier et al., 2021a). Most patients in this study sample were treated with biological therapies, which increases the relevance of the obtained results since they might be treated with biosimilars in the future.

Most patients in the study sample had not heard of biosimilar medicines before. This is not surprising in view of the limited biosimilar market shares in the retail setting and demonstrates the knowledge gap among Belgian patients (Moorkens et al., 2020c; Vandenplas et al., 2021b). When looking at those patients that are familiar with biosimilars, they were mainly informed by their physician, patient association, and the internet. This is in accordance with the earlier findings of van Overbeeke et al. among Belgian patients with RA (van Overbeeke et al., 2017). Yet, a greater importance of physicians and the internet as the predominant sources was observed in this study.

Approximately half of the patients that heard of biosimilars before were aware that biosimilars have equivalent clinical outcomes compared to its reference biological product. The remaining patients selected an incorrect statement or indicated that they did not know exactly what biosimilars were, despite having heard of them. This further emphasizes the existing knowledge gap and the need for tailored educational programs for Belgian patients. No significant differences were found within this sample between Dutch- and French-speaking patients or between higher and lower educated patients.

### 4.2 Perceptions and Opinions About Biosimilars

Among Belgian patients being treated with originator biologicals, an overall positive attitude was observed towards transitioning their therapy to a biosimilar medicine. Patients are willing to transition their therapy if their physician prescribes and explains it to them, with the support of their physician, pharmacist, and nurse throughout the process. This observation contrasts with the reluctance of patients generally suggested by Belgian physicians (Dylst et al., 2014; Moorkens et al., 2020c; Vandenplas et al., 2020). The patient’s reluctance to switch to a biosimilar is often linked to the efforts HCPs have to make before patients can be convinced to switch. However, these additional efforts appear to be exaggerated in Belgium from the physicians’ point of view, based on this study. Nonetheless, even if patients are willing to switch, additional efforts still remain for HCPs in terms of training patients with the new injection device for SC products and organizing a consultation moment to introduce and explain the switch to the patient. Policy measures designed to compensate for the additional workload of healthcare providers when switching (i.e. gainsharing or benefit-sharing), as previously done in Belgium in 2019 among physicians in the ambulatory care, miss their target and ignore the need for a broader approach if they are targeted at the individual physician. Such incentives with the goal of supporting biosimilar use fail their purpose because they are directed only towards the physician. They do not support transitioning itself and ignore the multidisciplinary nature of transitioning, which involves other HCPs as well. In addition, such an incentive does not add value from the patient’s perspective. A recent study showed that the Belgian healthcare providers' knowledge of biosimilar medicines is low (Barbier et al., 2021b). Hence, there is a significant challenge to properly train caregivers in the first place, either during their pre-graduate training or as continued education. Belgian HCPs must first of all be properly trained to adequately guide patients, so they can provide the support that patients expect from them when transitioning to a biosimilar. In addition, adequate compensation from the government under the form of gainsharing is needed. Gainsharing schemes should benefit the needs within that therapeutic domain in close consultation with the relevant physician associations. In this way an incentive is created whereby both HCPs and patients experience the added value of using biosimilars.

In terms of willingness to transitioning and possible conditions, no significant differences were found between higher and lower educated people. Yet, the results do suggest that Dutch-speaking patients have higher demands for a possible transitioning to a biosimilar. Significantly more Dutch-speaking patients wanted the biosimilar to be tested in their disease area before switching, as well as only if they were not satisfied with their current originator therapy.

In 2018, the Belgian regulatory agency and the national health insurer collaborated on an educational campaign to inform the wider public about biological medicines, with specific attention for biosimilars (Federaal Agentschap voor Geneemiddelen en Gezondheidsproducten, 2018). However, the impact of this campaign was rather low because it probably did not reach the patients sufficiently (Moorkens et al., 2020b). This is not surprising, because this study showed that patients who need information about biosimilars do not want such information via governmental agencies. They rather prefer to receive this via their physician, pharmacist, or patient organization. Moreover, the Belgian media campaign did not aim to reach out to the specific subgroup of patients looking for information. Instead, educational material was distributed via their website, radio, and folders or flyers. Future similar initiatives should be set up with a clear targeted communication strategy in close collaboration with patient associations and healthcare providers, who can always refer patients back to the right sources on the internet. Broader informational campaigns should not aim to target the wider public, but aim to target those patients who will need such information.

The existing knowledge gap among patients could be further enlarged due to the spread of negative or false information about biosimilars. This is one of the main reasons why it is essential to provide correct and unbiased information to patients, and to refer patients back to correct and reliable information (Cohen and McCabe, 2020; Vandenplas et al., 2021a). This study showed that Belgian patients will look for further information about biosimilars on the internet, a place where they might encounter false information. This information-seeking behavior of patients on the internet was recently linked to negative perceptions about transitioning to biosimilars (Gasteiger et al., 2020). Even patient organizations, who are one of the sources patients will preferably consult for information on biosimilars, must be careful not to spread negative or incorrect information (Vandenplas et al., 2021a). It is therefore the task of HCPs and patient associations to refer patients to trustworthy sources of information, avoiding misperceptions about biosimilars among the patient population.

### 4.3 Study Strengths and Limitations

This study has several strengths and limitations regarding its methods and design. The large sample size, with a representative sample of different therapeutic areas, regions, and age groups, depict a clear picture of the group of Belgian patients of interest for this research question. Flemish people might slightly be overrepresented, but they are also in the majority among the Belgian population (Statbel, 2021b). Moreover, as already discussed above, one should not focus on the knowledge and perceptions of all patients. It is of particular interest to measure these among patients who are currently treated with biological medicines. After all, these are the patients that could come into contact with biosimilars in the near future. The large proportion of patients in the study sample that are currently being treated with biologicals increases the relevance of the obtained results. This is the first study to assess the views of Belgian patients among all relevant therapeutic areas in the ambulatory care.

Patients were recruited through patient organizations. This could possibly have led to selection bias in the sample, since mostly patients affiliated to a patient association participated in this study. Possibly, the level of knowledge among Belgian patients affiliated to patient organizations is higher than in the overall population (Jacobs et al., 2016). In addition, previous research has revealed that a better knowledge about biosimilars leads to higher acceptance and better clinical outcomes (Tweehuysen et al., 2018a; 2018b). As a result, the overall positive perception among the study population might be overestimated. Moreover, the researchers chose to assess the knowledge and perceptions of patients via online questionnaires. Because the survey could only be completed online, participation required access to the internet. However, not every Belgian patient is connected to the internet, so this group was excluded for participation. Since patient associations were responsible for reaching patients (via newsletters, website, social media), no response rate could be calculated.

The proportion of higher educated people was higher than one would expect to represent the entire Belgian population (Statbel, 2021a). Possibly, the topic of the survey deterred people with lower health literacy, as this group is presumably larger among less educated people (van der Heide et al., 2013). An earlier report by the Belgian Healthcare Knowledge Center (KCE) revealed that approximately 40% of the Belgian population has low or insufficient health literacy levels (KCE, 2020). This means that possibly an important subgroup was not sufficiently represented in this study. This might have resulted in a slight overestimation of the level of knowledge and positive perceptions about biosimilars. Nonetheless, we did not find any statistically significant differences between higher and lower educated patients on their perceptions or knowledge about biosimilars in this sample (Cfr. Supplementary Material).

### 4.4 Avenues for Further Research

Much research has already been conducted regarding informing patients about biosimilars in recent years. However, what still remains unclear is which specific communication strategies are most successful in increasing patient knowledge or trust. An important question here is also whether increasing knowledge can be causally linked to higher acceptance by patients. Future research could investigate to what extent educational initiatives reach patients, as well as to what extent they contribute to greater acceptance of biosimilars. Additional research should also be conducted on how to reach patients and which channels are most efficient for each specific subgroup. Presumably, a different approach is needed in how to reach patients with lower health literacy with accurate information about their health, and in particular medicines.

## 5 Conclusion

Biosimilar medicines are equal treatment alternatives for existing off-patent originator biologicals and may generate competition in the Belgian off-patent biologicals market. Despite their market entry already several years ago, most biosimilar medicines have low market shares in the Belgian ambulatory care setting. One of the suggested reasons for this is the lack of trust in biosimilars among Belgian patients, leading to low acceptance and limited uptake in clinical practice. However, despite the existing knowledge gap, this study showed that most Belgian patients treated with originator biologicals are willing to transition to a biosimilar in the future. Patients expect support from all stakeholders when transitioning to biosimilars. In particular, HCPs and patient associations have a key role when informing patients as the trusted sources. In order to build trust in biosimilar medicines among Belgian patients, initiatives to educate patients should go hand in hand with HCP education and collaboration with patient organizations. Further investments in informing patients about biosimilars may contribute to a more affordable Belgian healthcare system.

## Data Availability

The raw data supporting the conclusions of this article will be made available by the authors, without undue reservation.
