# Supplementary material for: Perceptions About Biosimilar Medicines Among Belgian Patients in the Ambulatory Care
Source: Front Pharmacol. 2022 Jan 5;12:789640. doi: 10.3389/fphar.2021.789640 (PMC8766979; doi:10.3389/fphar.2021.789640)
Supplement: Supplementary file 1 [file DataSheet1.PDF]

Supplementary Material

Perceptions about biosimilar medicines among Belgian patients in the ambulatory care

Yannick Vandenplas[1+], Liese Barbier[1], Steven Simoens[1], Philippe Van Wilder[2], Arnold G. Vulto[1,3], Isabelle Huys[1]

*** Correspondence:**
Yannick Vandenplas
yannick.vandenplas@kuleuven.be

Supplementary files

S1: Available biosimilar products in the Belgian ambulatory care setting (September 2021)

S2: Questionnaire (English)

S3: Additional statistical analyses to assess differences between high and low educated patients, and Dutch-speaking versus French-speaking.

S1: Available biosimilar products in the Belgian ambulatory care setting (September 2021)

Molecule	Biosimilar product	Date of reimbursement
**Adalimumab**	Amgevita<br>Imraldi<br>Hyrimoz<br>Hulio<br>Idacio	March 2017<br>August 2017<br>July 2018<br>September 2018<br>April 2019
**Etanercept**	Benepali<br>Erelzi<br>Nepexto	January 2016<br>July 2019<br>February 2021
**Insulin glargine**	Abasaglar	June 2016
**Enoxaparin sodium**	Ghemaxan	January 2021
**Follitropin-alpha**	Ovaleap	September 2013
**Filgrastim**	Tevagrastim<br>Nivestim<br>Accofil	September 2008<br>June 2010<br>September 2014
**Insulin lispro**	None available in Belgium	
**Teriparatide**	None available in Belgium	

S2: Questionnaire (English)

Demographics

Please fill out the information below. We would like to collect your answers on the questions below to learn more about you. The answers of all participants on these questions will be summarized as group characteristics in reports and publications.

We would like to ask you to only fill in this questionnaire once.

1. What is your age?

   ☐ 18-29 years

   ☐ 30-39 years

   ☐ 40-49 years

   ☐ 50-59 years

   ☐ 60 years or more

2. What is your gender?

   ☐ Male

   ☐ Female

   ☐ Other

   ☐ I prefer not to answer

3. Where do you live?

   ☐ Flanders

   ☐ Wallonia

   ☐ Brussels

4. What type of disease are you diagnosed with? (multiple answers are possible)

- ☐ Plaque psoriasis or psoriasis vulgaris
- ☐ Psoriatic arthritis
- ☐ Diabetes Mellitus
- ☐ Rheumatoid arthritis
- ☐ Spondylitis ankylosans
- ☐ Crohn’s disease
- ☐ Ulcerative colitis
- ☐ Other: ________________

5. What is your highest education level?

- ☐ No diploma or primary school
- ☐ Secondary school
- ☐ Non-university higher education
- ☐ University higher education

Extra information for question 6:

*A **topical or local treatment** is a medical treatment directly applied to the skin. For example, by means of a cream, ointment. UV-therapy also falls under topical treatments.*

*An **oral treatment** means that your medicine is taken through the mouth. Several ways exist to administer a treatment orally, for example tablets, pills, capsules, syrup, or solutions.*

***Biological treatments** are administered directly into the human body. This can be intravenous or subcutaneous (via a pen, syringe).*

6. What is/are your current treatment(s) for your disease (only treatment(s) related the disease of interest for this study)? (multiple answers are allowed)

- ☐ Local (topical) treatment(s)

☐ Oral treatment(s)

☐ Biological treatment (One of the following therapies: etanercept, infliximab, adalimumab, certolizumab pegol, golimumab, abatacept, tocilizumab, guselkumab, risankizumab, brodalimumab, ixekizumab, secukinumab, ustekinumab, sarilumab, vedolizumab, insulin)

☐ Other: ____________________

Knowledge and awareness

7. Have you ever heard of biosimilar medicines before?

☐ Yes

☐ No

8. If yes, through what source were you informed about biosimilars? (multiple answers are possible)

☐ Physician

☐ Other medical staff (pharmacist, nurse etc)

☐ Patient organization

☐ Work/education

☐ Social media (Facebook, Twitter, Instagram, etc)

☐ Searching on the internet

☐ Other: __________________

9. Which of the following statement(s) is correct regarding biosimilar medicines? (multiple answers are possible)

A biosimilar:

- ☐ Is structurally identical to the original biological medicine (the reference product)
- ☐ Is equivalent in terms of quality, safety, and efficacy to the original biological product
- ☐ Is similar to the original biological medicines, but differences in quality, safety, and efficacy exist
- ☐ Is a medicine which is authorized based on pharmacokinetic bio-equivalence with the original biological medicine
- ☐ Is a medicine that is authorized based on more extensive data (such as clinical data), than the data for generic medicines
- ☐ I heard about biosimilars before, but I don’t know what it means exactly
- ☐ I have never heard of biosimilars before

10. Indicate what is correct in your opinion about biosimilar medicines.

A biosimilar is:

- ☐ As effective and safe compared to its original biological medicine
- ☐ More effective and safe compared to its original biological medicine
- ☐ Less effective and safe compared to its original biological medicine
- ☐ I don’t know

Perceptions

Please read carefully both definitions below before you proceed to the following questions.

Definition of biosimilar medicines:

A biosimilar medicine ("biosimilar") is a biological medicine that is highly similar to an existing biological medicine already placed on the EU market (the so-called 'biological reference medicine'). Pharmaceutical companies may market approved biosimilars at the end of the market protection period of the reference biological medicine.

Due to the natural variability and complex production methods of biological medicines, it is not possible to exactly copy the original reference medicine. Minor differences may be present between the biosimilar and the reference medicine. A biosimilar is therefore not considered a generic of a biological medicine. However, strict controls are carried out during the manufacturing process to ensure that any minor differences do not affect the way the medicine works or its safety. These differences are therefore of no clinical significance in terms of safety or efficacy.

Definition of generic medicines:

A generic medicine is a medicine which is medicine that contains the same active substance a medicine that is already authorized on the market. Generics are traditional medicines that are chemically synthesized (e.g. aspirin, paracetamol), and are not prone to inherent variability like biological medicines. Pharmaceutical companies are allowed to market generics when market protections have expired on the original product. In contrast to biosimilar medicines, generics are structurally identical to their original medicine.

Definition of interchangeability:

Interchangeability refers to the ability to exchange one drug for another that is expected to have the same clinical effect. This means that your physician can make the decision with the patient to change or switch current biological treatment with its biosimilar.

11. When you are as a patient being treated with an original **biological** medicine. Do you think your treating **physician** should be able to change the current biological therapy with its biosimilar?

☐ Yes

☐ No

☐ Depending on the product class

☐ Other: __________

12. When you are as a patient being treated with an original **biological** medicine. Do you think your **pharmacist** should be able to change the current biological therapy with its biosimilar?

☐ Yes

☐ No

☐ Depending on the product class

☐ Other: __________

13. When you are as a patient being treated with an original **chemical** medicine. Do you think your treating **physician** should be able to change the current therapy with its generic?

☐ Yes

☐ No

☐ Depending on the product class

☐ Other: ____________________________________

14. When you are as a patient being treated with an original **chemical** medicine. Do you think your **pharmacist** should be able to change the current therapy with its generic?

☐ Yes

☐ No

☐ Depending on the product class

☐ Other: ____________________________________

15. Are you currently being treated with a **biological** medicine?

- ☐ Yes, with an original biological medicine
- ☐ Yes, with a biosimilar medicine
- ☐ Yes, but I don’t know whether it is an original biological or a biosimilar medicine
- ☐ I don’t know
- ☐ No

16. Under what circumstances would you change your original biological therapy with its biosimilar medicine? (multiple answers are possible)

- ☐ If my physician prescribes the biosimilar and supports this decision
- ☐ When the biosimilar has been tested in patients with my disease
- ☐ When the biosimilar is cheaper for the Belgian healthcare system than my current originator treatment
- ☐ When the biosimilar is cheaper for me than my current originator treatment
- ☐ When I would not be satisfied with my current treatment
- ☐ When I would be stable on my current treatment
- ☐ When the injection device would be easier/improved compared to my current biological medicine
- ☐ Never
- ☐ Other: ______________________

17. Which questions would you ask when your treating physician proposes you to change your current biological therapy with it biosimilar? (multiple answers are possible)

- ☐ About side effects and efficacy
- ☐ About evidence from clinical trials
- ☐ About the reason why the transition is being made
- ☐ To change back to my original biological therapy
- ☐ About what are the physicians’ experiences with changing originator biological therapies to biosimilars
- ☐ I would agree without further questions
- ☐ Other: ______________________

18. What kind of support do you expect when the decision has been made to change your current biological therapy with its biosimilar?

- ☐ My physician has to explain this decision and inform me
- ☐ My physician can take this decision without informing me
- ☐ My physician should explain possible differences in injection devices
- ☐ My pharmacist has to be able to respond to my questions or concerns about biosimilars
- ☐ My pharmacist should explain possible differences in injection devices
- ☐ A nurse has to be able to respond to my questions or concerns about biosimilars
- ☐ A nurse should explain possible differences in injection devices
- ☐ I would like to receive brochures, leaflets, or other informational material about biosimilars from my physician or pharmacist
- ☐ I would look for more information about biosimilars on the internet myself
- ☐ Other: _________________

19. When I want to know more about biosimilar medicines, I would:

- ☐ Ask my physician
- ☐ Ask my pharmacist
- ☐ Search for information on the internet
- ☐ Search for information via social media (Facebook, Twitter, Instagram, etc)
- ☐ Ask my patient organization or other patients within my patient organization
- ☐ Other: _____________________

20. Whom do you consider a trusted source of information about **medicines in general**? (multiple answers are possible)

- ☐ Patient association
- ☐ Physician
- ☐ Pharmacist
- ☐ Nurse
- ☐ Pharmaceutical industry
- ☐ Academia (universities)
- ☐ Regulatory authority (FAMHP, EMA)
- ☐ National health insurer (NIHDI) or mutualities
- ☐ Ministry of Health/Social Affairs
- ☐ Other: _________________

21. Whom do you consider a trusted source of information about **biosimilar medicines**? (multiple answers are possible)

- ☐ Patient organization
- ☐ Physician
- ☐ Pharmacist
- ☐ Nurse
- ☐ Pharmaceutical industry
- ☐ Academia (universities)
- ☐ Regulatory authority (FAMHP, EMA)
- ☐ National health insurer (NIHDI) or mutualities
- ☐ Ministry of Health/Social Affairs
- ☐ Other: _________________

22. What kind of information would you like to receive about biosimilar medicines as a patient?

Information about:

- ☐ Clinical development process of the biosimilar (for example, in which patient groups the biosimilar has been tested)
- ☐ General development process of a biosimilar
- ☐ Safety of the biosimilar
- ☐ Price and reimbursement of biosimilars in Belgium
- ☐ How biosimilars are evaluated and approved in Europe
- ☐ Quality requirements of biosimilars
- ☐ Societal cost and possible savings of biosimilars in Belgium
- ☐ Information about the injection device of the biosimilar
- ☐ I don't need any information about biosimilars
- ☐ Other: _____________________

Ending

Thank you for your participation to this study. Your contribution is valued and will contribute to our understanding about the patient perspective regarding biological and biosimilar medicines.

If you have any further comments related to this study, or related to the broader context of this topic, please include them in the text box below.: ________________

S3: Additional statistical analyses and corresponding figures

Table S1: Statistical analysis to assess differences between higher and lower educated patients	
Question: Under what circumstances would you change your current original biological therapy with its biosimilar?	
	Fisher Exact test (two tailed)*
	p-value**
**Statement 1:** If my physician prescribes the biosimilar	p=0,14894
**Statement 2:** When the biosimilar had been tested in patients with my disease	p=0,13787
**Statement 3:** When I would not be satisfied with my current treatment	p=0,58204
**Statement 4:** When the biosimilar is cheaper for the Belgian healthcare system than my current treatment	p=0,67348
**Statement 5:** When the injection device would be better than my current treatment	p=0,45342
**Statement 6:** When the biosimilar is cheaper for me than my current treatment	p=0,11963
**Statement 7:** When I would be stable under my current treatment	p=0,73167
**Statement 8:** Never	p=0,68709
*Answers of respondents with higher (N = 167) versus lower (N = 82) education were compared.	
** p-values lower than 0,05 were considered statistically significant	

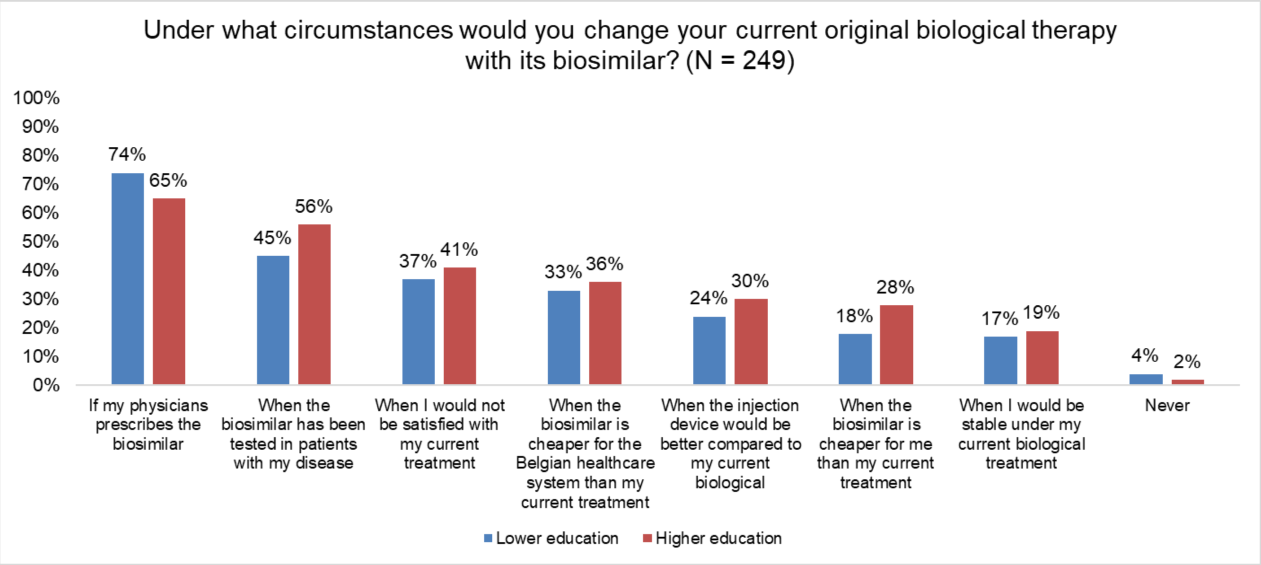

**Table S2: Statistical analysis to assess differences between Dutch-speaking and French-speaking patients**	
**Question: Under what circumstances would you change your current original biological therapy with its biosimilar?**	
	**Fisher Exact test (two tailed)***
	**p-value****
**Statement 1:** If my physician prescribes the biosimilar	p=0,77718
**Statement 2:** When the biosimilar had been tested in patients with my disease	p=0,00142
**Statement 3:** When I would not be satisfied with my current treatment	p=0,00698
**Statement 4:** When the biosimilar is cheaper for the Belgian healthcare system than my current treatment	p=0,58208
**Statement 5:** When the injection device would be better than my current treatment	p=0,30317
**Statement 6:** When the biosimilar is cheaper for me than my current treatment	p=0,44360
**Statement 7:** When I would be stable under my current treatment	p=0,73385
**Statement 8:** Never	p=0,70294
*Answers of Dutch-speaking (N = 160) versus French-speaking (N = 89) patients were compared.	
** p-values lower than 0,05 were considered statistically significant	

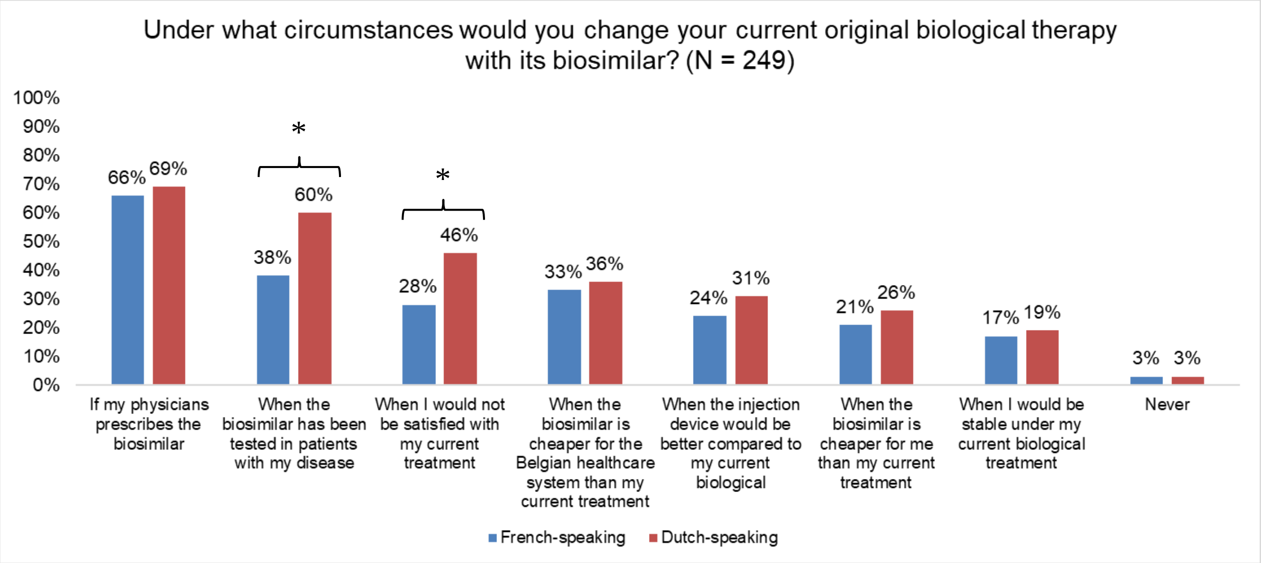

* indicates that a significant difference (p < 0,05) was found

**Table S3: Statistical analysis to assess differences between higher and lower educated patients**	
**Question: Indicate what is correct about biosimilar medicines in your opinion. A biosimilar is:**	
	**Fisher Exact test (two tailed)***
	**p-value****
**Statement 1:** As effective and safe compared to its original biological medicine	p=0,13464
**Statement 2:** More effective and safe compared to its original biological medicine	p=1,0000
**Statement 3:** Less effective and safe compared to its original biological medicine	p=0,30088
**Statement 4:** I don’t know	p=0,25189
*Answers of respondents with higher (N = 167) versus lower (N = 82) education were compared.	
** p-values lower than 0,05 were considered statistically significant	

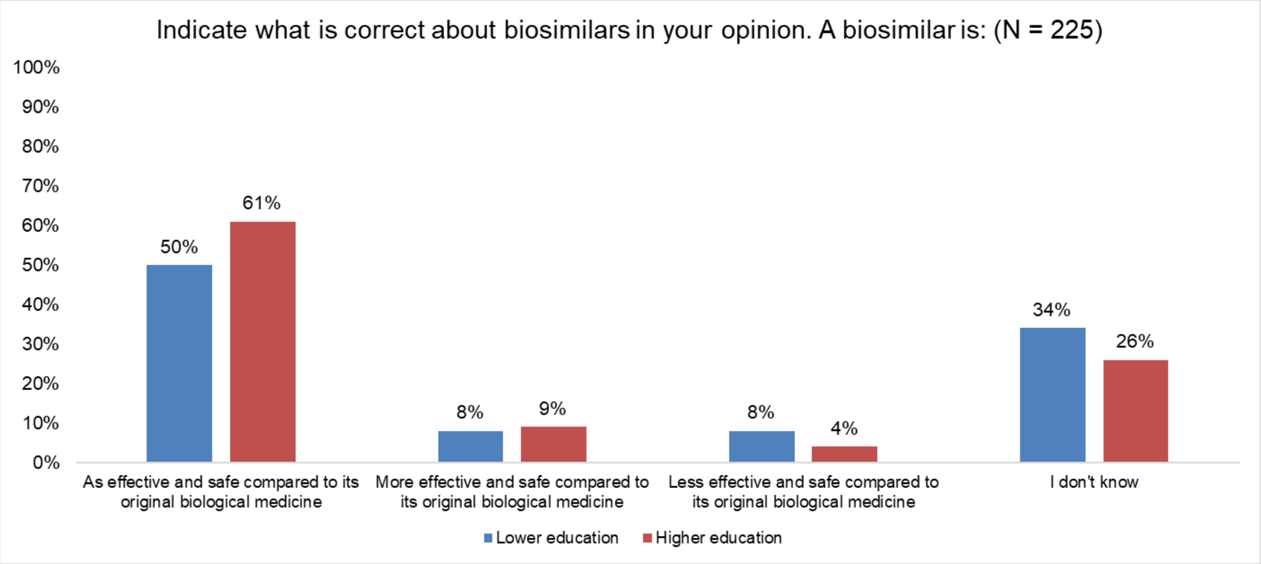

Table S4: Statistical analysis to assess differences between Dutch-speaking and French-speaking patients	
**Question: Indicate what is correct about biosimilar medicines in your opinion. A biosimilar is:**	
	**Fisher Exact test (two tailed)***
	**p-value****
**Statement 1:** If my physician prescribes the biosimilar	p=0,76504
**Statement 2:** When the biosimilar had been tested in patients with my disease	p=0,28912
**Statement 3:** When I would not be satisfied with my current treatment	p=1,0000
**Statement 4:** When the biosimilar is cheaper for the Belgian healthcare system than my current treatment	p=0,25189
* Answers of Dutch-speaking (N = 160) versus French-speaking (N = 89) patients were compared.	
** p-values lower than 0,05 were considered statistically significant	

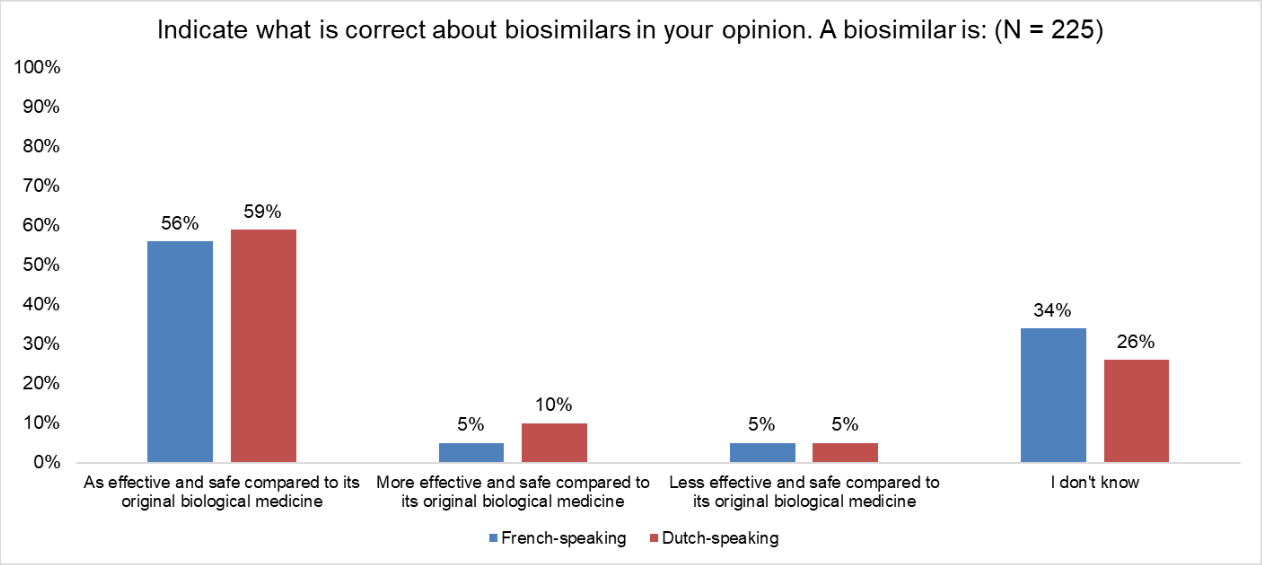
Indicate what is correct about biosimilars in your opinion. A biosimilar is: (N = 225)
100%
90%
80%
70%
60%
50%
40%
30%
20%
10%
0%
56%
59%
5%
10%
5%
5%
34%
26%
As effective and safe compared to its original biological medicine
More effective and safe compared to its original biological medicine
Less effective and safe compared to its original biological medicine
I don't know
French-speaking
Dutch-speaking
